# Pior Prognóstico de Pacientes com IAMCST Fora do Horário de Expediente: O que Estamos Perdendo?

**DOI:** 10.36660/abc.20240701

**Published:** 2025-02-06

**Authors:** Marco Antônio Smiderle Gelain, Henrique Barbosa Ribeiro

**Affiliations:** 1 Hospital das Clínicas da Faculdade de Medicina da Universidade de São Paulo Instituto do Coração São Paulo SP Brasil Instituto do Coração do Hospital das Clínicas da Faculdade de Medicina da Universidade de São Paulo, São Paulo, SP – Brasil; 2 Hospital Sírio-Libanês São Paulo SP Brasil Hospital Sírio-Libanês, São Paulo, SP – Brasil

**Keywords:** IAMCSST, Síndrome Coronariana Aguda, Fora do Horário de Expediente

No Brasil, a incidência anual de infarto do miocárdio é estimada em aproximadamente 300.000 a 400.000 casos.^1^ Apesar dos avanços no tratamento, o infarto do miocárdio continua sendo a principal causa de morte no Brasil e no mundo,^2^ com uma taxa de letalidade em torno de 9%.^3^ De acordo com o registro BRACE, a síndrome coronariana aguda (SCA) é responsável por 45% das hospitalizações no Brasil, com dois terços atribuídos ao infarto agudo do miocárdio (IAM), incluindo infarto do miocárdio com supradesnivelamento do segmento ST (IAMCSST) e infarto do miocárdio sem supradesnivelamento do segmento ST (IAMSSST).^4^ Notadamente, 40% a 60% dos pacientes com IAMCSST se apresentam fora do horário de expediente, entre às 19h e às 7h, e nos fins de semana.^5,6^ Dado que o tratamento do IAMCSST é tempo-sensível^7^ e requer uma equipe multidisciplinar complexa no laboratório de cateterismo, o impacto do tempo de apresentação nos resultados do paciente continua sendo uma questão controversa. Assim, a questão de se a apresentação fora do horário de expediente resulta em um prognóstico pior ainda é questionável.

Nesta edição da revista, Cirne et al.^8^ examinaram um registro prospectivo de um centro de SCA de alto volume no sul do Brasil, abrangendo 4.436 pacientes consecutivos com IAMCSST submetidos à intervenção coronária percutânea primária (ICPP) entre 2009 e 2019. Os pacientes foram estratificados por tempo de apresentação: no horário de expediente versus fora do horário de expediente. O desfecho primário foi um composto de eventos cardíacos adversos maiores (ECAM), que incluiu morte, IAM ou acidente vascular cerebral em um ano, juntamente com ECAM em 30 dias, trombose de stent e necessidade de nova revascularização. Entre a coorte, 2.576 (57%) pacientes foram tratados fora do horário de expediente, sem diferenças significativas entre os grupos em relação à idade, sexo, artéria relacionada ao infarto ou classificação de Killip. Além disso, o tempo sintoma-hospital (média de 240 minutos) e o tempo porta-balão (TPB) (média de 70 minutos) foram semelhantes. Não foram observadas diferenças na complexidade do procedimento, complicações, uso de balão intra-aórtico ou taxas de sucesso de angioplastia; entretanto, pacientes fora do horário apresentaram uma carga trombótica maior (49,6% vs. 45,5%, p<0,01). Apesar das características clínicas e angiográficas basais comparáveis, aqueles que se apresentaram fora do horário apresentaram maiores taxas de ECAM em 30 dias e um ano (10,2% vs. 8,5% e 15,4% vs. 13,1%, respectivamente), bem como maior mortalidade (hospitalar: 7,9% vs. 6,1%; 30 dias: 7,8% vs. 6,1%; 1 ano: 11,1% vs. 9%, respectivamente). Não foram observadas diferenças na incidência de IAM ou AVC.

**Figura 1:**
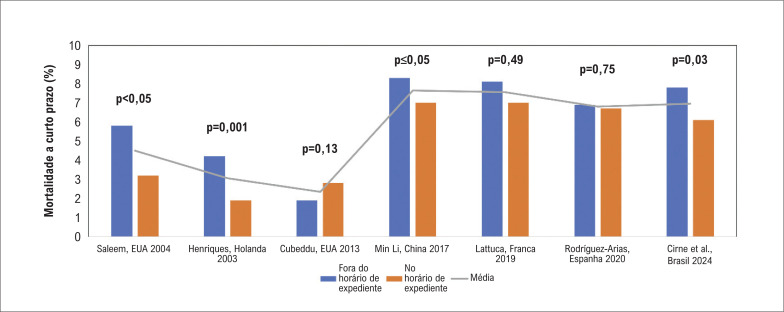
Taxas de mortalidade de curto prazo em pacientes com IAMCST fora do horário de expediente vs. dentro do horário de expediente.

Desde o início dos anos 2000, a relação entre o TPB e o aumento da mortalidade em pacientes com IAMCSST^9^ gerou preocupações quanto ao "efeito fim de semana", sugerindo que os pacientes tratados fora do horário de expediente tendem a apresentar resultados piores.^10^ Numerosos estudos e meta-análises produziram resultados conflitantes sobre o risco de ECAM durante o acompanhamento de curto e longo prazo.^5,6,11,12^ Notadamente, uma meta-análise de 2017^13^ envolvendo 192.658 pacientes indicaram mortalidade elevada a curto prazo para apresentações fora do horário de expediente, embora esse prognóstico adverso tenha diminuído para pacientes submetidos a ICPP. Na última década, vários estudos de países desenvolvidos mostraram que as disparidades nos resultados para pacientes fora do horário de expediente diminuíram.^5,6,14–16^ Os fatores contribuintes podem incluir melhorias nos processos de atendimento, treinamento aprimorado da equipe e distribuição mais equitativa de recursos 24 horas por dia. Essa tendência é resumida na Figura 1, que destaca dados de estudos importantes nos últimos 20 anos, confirmando a ausência de diferenças significativas nos resultados para apresentações de IAMCSST em horário de expediente versus fora do horário de expediente. No entanto, alguns centros ainda relatam resultados piores para pacientes fora do horário de expediente.^17^

Vários aspectos do estudo de Cirne et al.^8^ merecem consideração adicional. Embora nenhum aumento estatisticamente significativo em ECAM tenha sido observado durante a hospitalização, um aumento notável na mortalidade foi evidente, persistindo durante o acompanhamento de médio prazo. Mesmo com tempo sintoma-hospital semelhante, esses achados sugerem um viés potencial na capacidade dos pacientes de reconhecer o início dos sintomas durante as horas noturnas, uma hipótese já proposta anteriormente.^6^ Isso poderia prolongar a duração isquêmica, como evidenciado pela maior carga trombótica e pelo uso aumentado de inibidores da glicoproteína IIb/IIIa em pacientes fora do horário, potencialmente exacerbando os resultados hospitalares relacionados à insuficiência cardíaca e suas complicações, que não foram relatados. Além disso, a qualidade diferencial do atendimento fora do horário — difícil de medir objetivamente — pode desempenhar um papel, pois o desempenho do médico e a disponibilidade de recursos podem ser comprometidos, apesar dos tempos porta-balão comparáveis entre os grupos. Também devemos considerar as taxas mais altas de ECAM não relacionadas a eventos isquêmicos após a alta hospitalar, sugerindo a influência potencial de variáveis não aferidas, como o desenvolvimento de insuficiência cardíaca, mortalidade não cardíaca, adesão irregular à medicação e acompanhamento inadequado após a alta.

Dados esses resultados, é imperativo buscar por um sistema de saúde que garanta uma qualidade uniforme de atendimento 24 horas por dia, 7 dias por semana, principalmente à luz da descoberta de que até 57% dos pacientes com IAMCSST se apresentam fora do horário de expediente. Também devemos priorizar o acompanhamento contínuo desses pacientes após a alta. Em última análise, o impacto do tempo de apresentação nos resultados do IAMCSST continua sendo uma questão não resolvida. Os insights de estudos como o de Cirne et al.^8^ são vitais para melhorar nossa compreensão e aperfeiçoar o atendimento a essa população de alto risco.
