# Supplementary material for: Pior Prognóstico de Pacientes com IAMCST Fora do Horário de Expediente: O que Estamos Perdendo?
Source: Arq Bras Cardiol. 2025 Feb 6;121(11):e20240701. [Article in Portuguese] doi: 10.36660/abc.20240701 (PMC11809846; doi:10.36660/abc.20240701)
Supplement: Translation

# Translation: Worse Prognosis of STEMI Patients during Off-Hours: What are we Missing?

**DOI:** 10.36660/abc.20240701i

**Authors:** Marco Antônio Smiderle Gelain, Henrique Barbosa Ribeiro

**Affiliations:** 1 São Paulo SP Brazil Instituto do Coração do Hospital das Clínicas da Faculdade de Medicina da Universidade de São Paulo, São Paulo, SP – Brazil; 2 São Paulo SP Brazil Hospital Sírio-Libanês, São Paulo, SP – Brazil

**Keywords:** STEMI, Acute Coronary Syndrome, Off-hours

In Brazil, the annual incidence of myocardial infarction is estimated at approximately 300,000 to 400,000 cases.^1^ Despite advancements in treatment, myocardial infarction remains the leading cause of death in Brazil and globally,^2^ with a lethality rate of around 9%.^3^ According to the BRACE registry, acute coronary syndrome (ACS) accounts for 45% of hospitalizations in Brazil, with two-thirds attributed to acute myocardial infarction (AMI), including both ST-segment elevation myocardial infarction (STEMI) and non-ST-segment elevation myocardial infarction (NSTEMI).^4^ Notably, 40% to 60% of STEMI patients present during off-hours, between 7 PM and 7 AM, and on weekends.^5,6^ Given that the treatment of STEMI is time-sensitive^7^ and requires a complex multidisciplinary team in the catheterization laboratory, the impact of presentation timing on patient outcomes remains a contentious issue. Thus, the question of whether off-hours presentation results in a poorer prognosis is still debated.

In this issue of the journal, Cirne et al.^8^ examined a prospective registry from a high-volume ACS center in Southern Brazil, encompassing 4,436 consecutive STEMI patients who underwent primary percutaneous coronary intervention (PPCI) between 2009 and 2019. Patients were stratified by presentation time: on-hours versus off-hours. The primary outcome was a composite of major adverse cardiac events (MACE), which included death, AMI, or stroke at one year, along with MACE at 30 days, stent thrombosis, and the need for new revascularization. Among the cohort, 2,576 (57%) patients were treated during off-hours, with no significant differences between the groups concerning age, sex, infarction-related artery, or Killip classification. Additionally, symptom-to-hospital time (mean 240 minutes) and door-to-balloon time (DTB) (mean 70 minutes) were similar. No differences were observed in procedural complexity, complications, intra-aortic balloon pump usage, or angioplasty success rates; however, off-hours patients exhibited a higher thrombotic burden (49.6% vs. 45.5%, p<0.01). Despite comparable baseline clinical and angiographic characteristics, those presenting off-hours had higher rates of MACE at 30 days and one year (10.2% vs. 8.5% and 15.4% vs. 13.1%, respectively), as well as increased mortality (in-hospital: 7.9% vs. 6.1%; 30 days: 7.8% vs. 6.1%; 1 year: 11.1% vs. 9%, respectively). No differences were noted in AMI or stroke incidence.

Since the early 2000s, the link between DTB and increased mortality in STEMI patients^9^ has prompted concerns regarding the "weekend effect," suggesting that patients treated during off-hours tend to experience worse outcomes.^10^ Numerous studies and meta-analyses have produced conflicting findings on the risk of MACE during short- and long-term follow-up.^5,6,11,12^ Notably, a 2017 meta-analysis^13^ involving 192,658 patients indicated heightened short-term mortality for off-hours presentations, though this adverse prognosis diminished for patients who underwent PPCI. Over the past decade, several studies from developed countries have shown that disparities in outcomes for off-hour patients have largely disappeared.^5,6,14–16^ Contributing factors may include improvements in care processes, enhanced staff training, and more equitable resource distribution around the clock. This trend is summarized in Figure 1, which highlights data from major pivotal studies over the last 20 years, confirming the absence of significant differences in outcomes for on- versus off-hour STEMI presentations. Nonetheless, some centers still report poorer outcomes for off-hour patients.^17^

**Figure 1:** Off-hours vs. on-hours short-term mortality rates in STEMI patients.

Several aspects of the study by Cirne et al.^8^ warrant further consideration. Although no statistically significant increase in MACE was observed during hospitalization, a notable rise in mortality was evident, persisting through mid-term follow-up. Even with similar symptom-to-hospital times, these findings suggest a potential bias in patients' ability to recognize the onset of symptoms during nighttime hours, a hypothesis previously proposed.^6^ This could prolong ischemic duration, as evidenced by the higher thrombotic burden and increased use of glycoprotein IIb/IIIa inhibitors in off-hour patients, potentially exacerbating in-hospital outcomes related to heart failure and its complications, which were not reported. Additionally, the differential quality of care during off-hours—difficult to measure objectively—may play a role, as physician performance and resource availability may be compromised despite comparable DTB times between groups. We must also consider the higher rates of MACE unrelated to ischemic events after hospital discharge, suggesting the potential influence of unmeasured variables such as the development of heart failure, non-cardiac mortality, irregular medication adherence, and inadequate post-discharge follow-up.

Given these results, it is imperative to strive for a healthcare system that ensures uniform quality of care 24/7, particularly in light of the finding that up to 57% of STEMI patients present during off-hours. We must also prioritize continuous follow-up for these patients post-discharge. Ultimately, the impact of presentation timing on STEMI outcomes remains an unresolved issue. The insights from studies like that of Cirne et al.^8^ are vital for enhancing our understanding and improving care for this high-risk population.
